# Rational Design of Materials Interface for Efficient Capture of Circulating Tumor Cells

**DOI:** 10.1002/advs.201500118

**Published:** 2015-07-16

**Authors:** Yong‐Qiang Li, Bevita K. Chandran, Chwee Teck Lim, Xiaodong Chen

**Affiliations:** ^1^ School of Materials Science and EngineeringNanyang Technological University50 Nanyang AvenueSIngapore639798Singapore; ^2^ School of Radiation Medicine and Protection and School for Radiological and Interdisciplinary Sciences (RAD‐X)Collaborative Innovation Center of Radiation Medicine of Jiangsu Higher Education InstitutionsMedical College of Soochow UniversitySuzhouJiangsu215123China; ^3^ Department of Biomedical EngineeringMechanobiology InstituteCentre for Advanced 2D MaterialsNational University of Singapore9 Engineering Drive 1Singapore117575Singapore

**Keywords:** circulating tumor cells, material interface, capture, tumor metastasis, cancer diagnosis

## Abstract

Originating from primary tumors and penetrating into blood circulation, circulating tumor cells (CTCs) play a vital role in understanding the biology of metastasis and have great potential for early cancer diagnosis, prognosis and personalized therapy. By exploiting the specific biophysical and biochemical properties of CTCs, various material interfaces have been developed for the capture and detection of CTCs from blood. However, due to the extremely low number of CTCs in peripheral blood, there exists a need to improve the efficiency and specificity of the CTC capture and detection. In this regard, a critical review of the numerous reports of advanced platforms for highly efficient and selective capture of CTCs, which have been spurred by recent advances in nanotechnology and microfabrication, is essential. This review gives an overview of unique biophysical and biochemical properties of CTCs, followed by a summary of the key material interfaces recently developed for improved CTC capture and detection, with focus on the use of microfluidics, nanostructured substrates, and miniaturized nuclear magnetic resonance‐based systems. Challenges and future perspectives in the design of material interfaces for capture and detection of CTCs in clinical applications are also discussed.

## 1 Introduction

Metastasis, the spread of tumor cells from the primary tumor site to vital distant organs through the circulatory system, is directly responsible for most carcinoma‐related deaths in cancer patients.^1^, ^2^, ^3^ Understanding the metastasis process and investigating the cause of metastasis will benefit the diagnosis and therapy of cancers, and have long been a focal point in the fight against malignant tumors.^4^, ^5^, ^6^ More than a century ago, Ashworth found tumor cells in the blood of an individual with metastatic cancer and suggested that these circulating tumor cells (CTCs) could originate from several tumors present in the patient.^7^ CTCs up to now have been found in patients with malignant tumors including lung, prostate, breast, colon and pancreatic cancers, but not in healthy individuals or patients with non‐malignant tumors. Hence, the relationship between the presence of CTCs and the development of metastases has been an important subject in tumor studies, and the number of CTCs is believed to be an important indicator of carcinoma progression and metastasis.^8^, ^9^, ^10^ Therefore, CTC enumeration can be used as a novel approach for cancer prognosis in which the enumeration values have been demonstrated to correlate to overall survival of patients with metastatic cancer. For example, patients with metastatic breast and prostate cancer have a lower survival rate if their CTC count is more than 5 CTCs per 7.5 mL of whole blood when using the CellSearch System.^11^ Compared to routine clinical analysis by collecting disseminated tumor cells via surgical removal or tumor biopsy, CTC enumeration from peripheral blood as a “liquid biopsy” is more convenient and amenable in practical operation.^12^ Furthermore, for patients undergoing cancer treatment, the decline of CTCs number is reported with the decrease of tumor size.^13^, ^14^ Hence, in addition to serving as a prognostic marker of cancer metastasis, CTC enumeration can also be used as a novel non‐invasive method to assess the efficacy of cancer therapeutic treatment and realize personalized therapy.

In recognition of the promising potential of CTCs in early cancer diagnosis and treatment, there is a growing interest in developing strategies for capture and detection of CTCs.^15^, ^16^, ^17^, ^18^ However, due to the extreme rarity of CTCs, with only 1–100 CTCs present in 1 mL of peripheral blood, which usually contains about five billion normal blood cells, CTC capture and subsequent detection are particularly challenging.^19^, ^20^ Currently, in term of the differences in biophysical and biochemical properties of CTCs as compared to normal blood cells, CTC capture strategies can be divided into two broad categories: biochemical methods and biophysical methods. Biochemical CTCs capture methods achieve selective CTCs isolation by affinity capture of unique biochemical markers expressed on surface of CTCs. For example, CellSearch system, a typical biochemical interface developed for CTC capture and isolation, is the first FDA‐approved system that processes 7.5 mL blood and enriches CTCs by the antibodies of CTCs unique biochemical marker of epithelial cell adhesion molecule (EpCAM) conjugated on magnetic beads, followed by microscopic cell imaging.^21^ Biophysical CTCs capture methods rely on differences in the physical properties of CTCs compared to normal blood cells such as cell size, deformability and density. Filtration and density gradient are two typical conventional biophysical methods for CTC capture and isolation.^22^, ^23^ Specifically, filtration provides size‐based separation of CTCs on the premise that they are larger than normal leukocytes and red blood cells, while density gradient centrifugation utilizes differences in cell density to separate CTCs from blood. Although CTC capture and isolation have been successfully achieved by these systems, the low CTC‐capture yield and purity of these systems are matters of concern.^24^, ^25^ Therefore, it is critical and urgent to develop some advanced material interfaces to achieve efficient capture and subsequent sensitive detection of rare CTCs for advancing biological and clinical cancer studies and applications.

**Figure 1:**
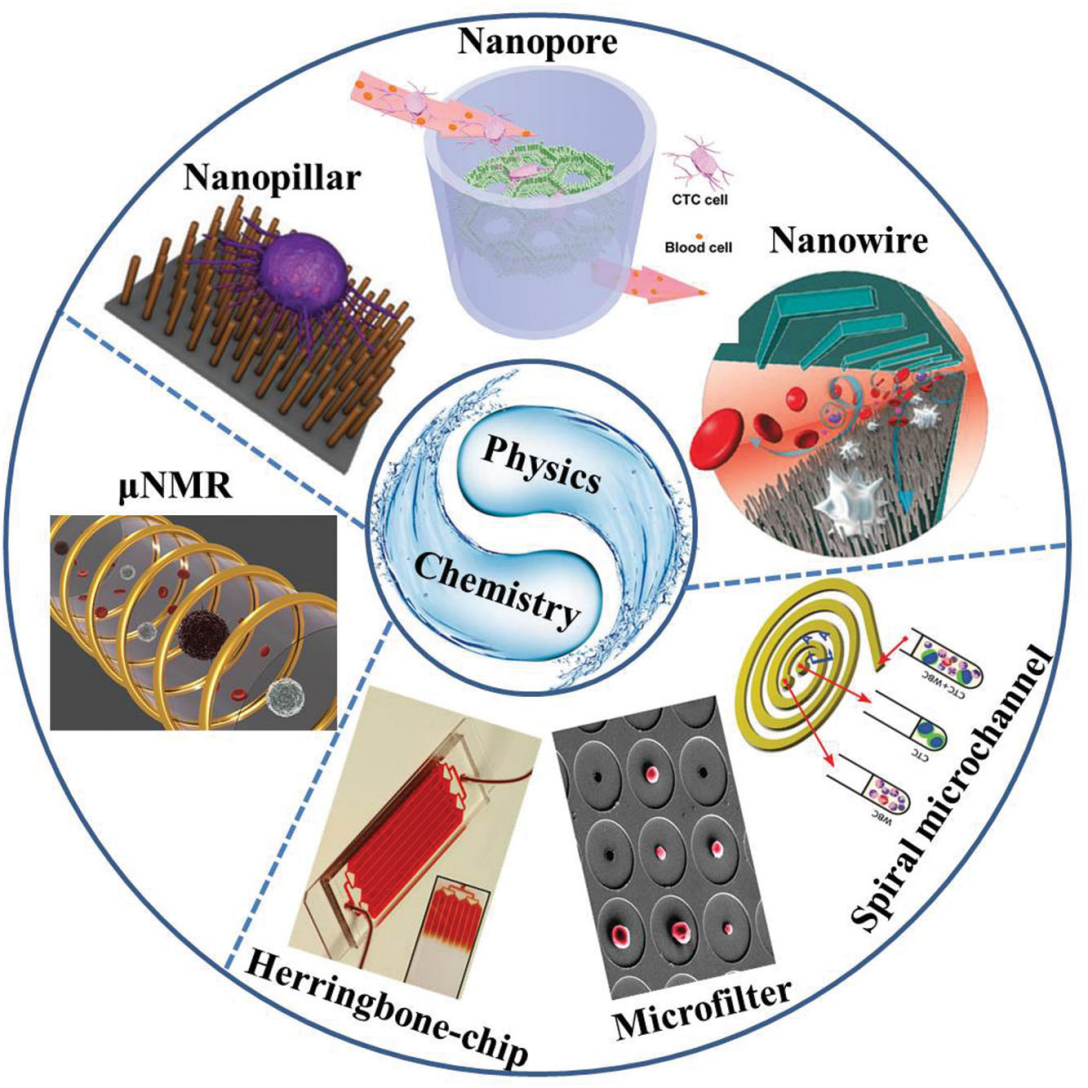
By exploiting the unique biophysical and biochemical properties of CTCs, various exquisite platforms for CTCs capture and detection have been designed, which mainly involve the microfluidic‐, nanostructured substrates‐, and μNMR‐based systems. Some representative examples are exhibited here. Reproduced with permission.^59^ Copyright 2010, American Chemical Society. Reproduced with permission.^66^ Copyright 2010, National Academy of Sciences. Reproduced with permission.^84^ Copyright 2012, Royal Society of Chemistry. Reproduced with permission.^94^, ^101^, ^122^

Recently, by exploiting the unique biophysical and biochemical properties of CTCs together with the development of nanotechnologies and advances in microfabrication and microfluidics, various exquisite material interfaces have been designed for outstanding capture and high‐sensitivity detection of rare CTCs (**Figure** **1**). In this review, we summarize recent representative works on the development of advanced material interfaces for CTC capture and detection. First, we will briefly introduce the known biophysical and biochemical properties of CTCs that can be employed for the design of these material interfaces. Subsequently, we will review the key advanced material interfaces, newly developed for efficient capture and detection of CTCs that can potentially revolutionize the future healthcare technology in cancer diagnosis and therapy, with focus on microfluidics, nanostructured substrates, and miniaturized nuclear magnetic resonance‐based systems. Lastly, we will present the challenges and future perspectives in the design of innovative materials interface for CTC capture and detection in clinical applications.

## 2 Biophysical and Biochemical Properties of CTCs

By presenting the possibility of being exploited to discriminate CTCs from normal blood cells, the biophysical and biomechanical properties of CTCs have gained much attention.^26^, ^27^, ^28^ In this section, we first review historical and recent studies of the biophysical properties of CTCs including density, size and deformability. Next, we present studies of the unique biochemical properties of CTCs concentrating on the specific surface receptors that can be used for the selective capture and isolation of CTCs from peripheral blood samples.

CTCs were first identified by Ashworth in 1869 when he microscopically inspected the blood from metastatic cancer patients. Due to their similarity to the metastatic cancer cells, CTCs identification were initially done by trained cytologists in term of elongated nuclei and fragmentation of the chromatin based on Papanicolaou's criteria for malignancy.^29^, ^30^ The density of CTCs were then investigated. Seal et al. studied the specific gravity of CTCs and leukocytes in density gradient centrifugation, and concluded that the specific gravity of CTCs was bigger than leukocytes and the method of density gradient centrifugation appeared to be a potential way for CTCs capture and isolation.^31^ In addition to the biophysical property of density, the tendency of CTCs to form clusters rather than individual cells was reported.^32^, ^33^, ^34^ With the development of microscopy and fluorescent staining technologies, more insight into the biophysical properties of CTCs including size and deformability were provided recently.^35^, ^36^ Several studies in breast cancer and lung adenocarcinoma noted that the size of CTCs was typically larger than blood cells, which has been an important criteria generally used for current CTCs capture and isolation.^37^, ^38^ In addition to size, cell deformability is another important biophysical property of CTCs frequently exploited in current capture and label‐free isolation of CTCs. Cell deformability refers to the ability of cells to change shape under a given level of applied stress without rupturing, which is an important biophysical property for CTCs to make them survive and transfer in the stressful environment of blood stream. Cell deformability can be indicated by the nuclear‐cytoplasmic ratio (NC ratio: the ratio of nuclear area and cell area with the nuclear area subtracted), where cells having larger NC ratio are likely to be less deformable. Meng et al. compared the average NC ratio of CTCs from 36 breast cancer patients with leukocytes, and concluded that CTCs always had larger NC ratio than leukocytes, which was consistent with the cell deformability data in which CTCs was less deformable than leukocytes.^39^ The decreased deformability of CTCs suggests that CTCs are stiffer than leukocytes, which provides an efficient way for CTCs capture and isolation from leukocytes.^40^ In summary, all the differences of biophysical properties between CTCs and leukocytes in term of density, size, internal structures and deformability demonstrated in these historical and recent studies, could be exploited for the label‐free isolation of CTCs.

In addition to the aforementioned biophysical properties, CTCs also express some unique biochemical markers that can be utilized for selective CTC capture and isolation. Among these biochemical markers, EpCAM and human epidermal growth factor receptor 2 (HER2) are two typical biochemical markers frequently used for the isolation and enrichment of CTCs. EpCAM is a transmembrane glycoprotein mediating Ca^2+^‐independent cell‐cell adhesion in epithelia.^41^, ^42^ EpCAM is found expressed on a great variety of human adenocarcinoma cells, but it is absent in blood cells.^43^ Hence, EpCAM is one expressed CTC‐associated biomarkers known, and CTC isolation techniques based on EpCAM antibodies are widely used.^44^, ^45^ The popular CellSearch system, which has been extensively used to capture and isolate CTCs from the blood of patients with cancers of the breast, prostate, and colon, employs a conjugation of EpCAM antibodies to ferrofluidic beads to enable the capture of CTCs through a magnetic field.^46^, ^47^, ^48^ In addition to EpCAM, several studies have reported that HER2 is overexpressed in CTCs of both metastatic and early breast cancer patients, and clinical data has shown that the change of HER2 status from low level expression to high level also occurred along with breast cancer recurrence and progression.^49^, ^50^ On the basis of this finding, HER2 is now considered to be a potential CTCs‐associated biomarker, and has also been widely used for CTC isolation and enrichment in clinical applications.

## 3 Microfluidics‐Based Material Interface for CTCs Capture

Based on the differences in biophysical properties between CTCs and normal blood cells described above, label‐free strategy for direct capture and isolation of CTCs can be developed. As a powerful separation approach, microfluidic technique with small sample‐volume requirement, fast processing times, multiplexing capabilities and large surface area‐to‐volume ratios, offer a good option for label‐free CTCs capture and isolation.^51^, ^52^ Recently, with the progress in nanobiotechnology and microfabrication, various microfluidic devices with rationally designed material interfaces have been developed for efficient CTCs capture, isolation and enrichment.^53^, ^54^, ^55^ These exquisite microfluidic systems will be briefly introduced in this section, and their performances on CTCs capture and isolation are summarized in **Table** **1**.

**Table 1:** The performances of microfluidic‐based platforms on CTCs capture and isolation

Microfluidic platform	Capture yield or efficiency	Detection rate or sensitivity	Cell survival rate	Reference
Microcavity filter	80%	–	98%	59
CTC‐chip	–	99%	–	17
Herringbone‐chip	91.8%	93%	95%	66
Arc‐shaped trap	89%	100%	–	80
Spiral microfluidics	80%	100%	–	84
Immunomagnetic microfluidic chip	90%	–	–	69

**Figure 2:**
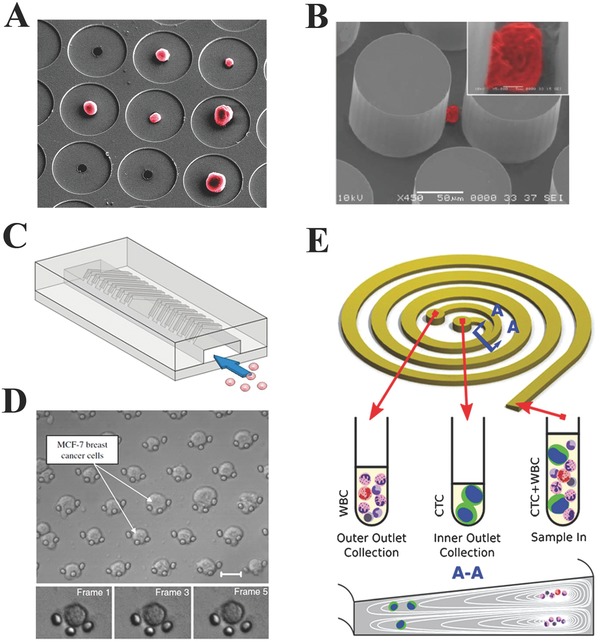
Evolution of the CTCs capture and enrichment methods based on microfluidics‐based material interfaces. A) Microcavity filter. Reproduced with permission.^59^ Copyright 2010, American Chemical Society. B) CTC‐chip. Reproduced with permission.^17^ Copyright 2007, Macmillan Publishers Ltd. C) Herringbone‐chip. Reproduced with permission.^66^ Copyright 2010, National Academy of Sciences. D) Arc‐shaped trap. Reproduced with permission.^80^ Copyright 2010, Elsevier. E) Spiral microfluidic channel. Reproduced with permission.^84^ Copyright 2012, Royal Society of Chemistry.

As a relatively straightforward technique with low cost, size‐based microfluidic filtration is one of the first approaches employed for CTCs capture and isolation, based on the fact that CTCs are larger than blood cells such as white blood cells (WBCs) and red blood cells (RBCs).^56^ Several membrane‐based microfilters have been developed for size‐based microfluidic capture and isolation of CTCs from peripheral blood samples.^57^, ^58^ Hosokawa et al. developed a microfluidic device equipped with a nickel microfilter for size‐based selective CTCs capture and isolation (**Figure** **2**A).^59^ In this microfluidic device, the nickel microfilter composed of 100×100 holes with the diameters between 8 and 11 μm was integrated between two PDMS funnels. Based on this microfluidic device, efficient isolation of CTCs from peripheral blood samples was obtained with high efficiency of greater than 80%. One advantage of this microfluidic device over conventional immunomagnetic CTCs separation platforms is its unique ability for efficient isolation of EpCAM‐negative CTCs. Moreover, approximately 98% of captured CTCs were found to be viable after fluorescent staining and washing processes. In another report, Vona et al. developed a polycarbonate microfilter‐based microfluidic device for size filtration of CTCs. The polycarbonate microfilter presented holes with diameter of 8 μm, and CTCs were efficiently trapped by this microflter while RBCs and WBCs with smaller sizes passed through it. Furthermore, this microfluidic system could run 12 samples in parallel and performed further identification and characterization of CTCs. In addition to above mentioned 2D microfilter‐based microfluidic devices, Zheng et al. recently fabricated a microfluidic system based on a microfilter with 3D pore structure for CTC isolation.^61^ The 3D microfilter was composed of two 10 μm thick Parylene C membranes separated by a 6.5 μm gap. Since the microholes fabricated on the top membrane were misaligned with that fabricated on the bottom membrane, large CTCs were efficiently trapped in this filter and finally, CTCs isolation from peripheral blood samples was successfully achieved based on this 3D microfilter‐based microfluidic platform. Moreover, a comparative study between the 2D and 3D microfilters was carried out, and it was found that the 2D microfilter might damage CTCs during the microfiltration process but the 3D microfilter could resolve this problem.

Additionally, microfluidic devices with functionalized microchannels have been developed for CTCs capture and isolation.^62^, ^63^, ^64^ Launiere et al. fabricated a microfluidic system with channels modified by alternating patterned biomimetic proteins (EpCAM antibody and E‐selectin) to increase target CTCs capture while reducing leukocyte's non‐specific adhesion by up to 82%.^62^ In addition to microfluidic platforms with barely immunoaffinity‐based strategy for CTCs capture, microfluidic platforms with functionalized microstructure arrays have also been developed for improved CTCs capture and isolation. Sheng et al. reported an aptamer‐functionalized microfluidic device with micropillar array for enhanced CTCs capture.^65^ This platform consisting of 59 000 micropillars, which could improve the interactions between CTCs and the aptamers, achieved efficient CTCs capture with efficiency of 95% from non‐processed whole blood samples. Nagrath et al. developed a unique microfluidic platform with EpCAM antibody‐coated microposts array for efficient and selective CTCs capture and isolation.^17^ Based on the selective interactions between target CTCs and the EpCAM antibody‐coated microposts, viable CTCs isolation from peripheral whole blood samples was achieved by this platform under precisely controlled laminar flow conditions. It was found that this platform successfully identified CTCs from peripheral whole blood samples of patients with different cancers with high sensitivity of 99% (Figure 2B). In addition to the functionalized micropost array‐based microfluidic platform, the same group also reported a herringbone‐based high‐throughput microfluidic mixing device (Herringbone‐Chip) for enhanced CTCs capture and isolation (Figure 2C). The Herringbone‐Chip consisted eight microchannels with patterned herringbone structures designed to generate microvortices and provide passive mixing of blood cells to enhance the interactions between CTCs and EpCAM antibody‐coated chip surface. Consequently, CTCs capture with efficiency of 91.8% was obtained based on this herringbone‐Chip in the blood samples prepared by spiking defined numbers of cancer cells into blood, as well as clinical blood samples from patients with metastatic disease, thereby indicating its great potential in clinical settings.^66^ Liu et al. developed a microfluidic devices with EpCAM antibody‐functionalized deterministic lateral displacement (DLD) chamber composed of triangular micropost arrays for CTCs capture.^67^ Based on the combination of microfluidic DLD array and high affinity‐based capture approach, efficient CTCs capture with 90% efficiency was obtained from spiked blood samples at low cell concentration (10^2^ cells mL^−1^). Kamande et al. reported a modular microfluidic system containing three different functional regions by which isolation, enumeration, and phenotyping of CTCs could be finished in one device.^68^

By combing the immunomagnetic separation strategy and microfluidic technique, Immunomagnetic‐based microfluidic systems have also been developed for efficient CTCs capture and isolation.^69^, ^70^, ^71^, ^72^, ^73^, ^74^, ^75^, ^76^ Hoshino et al. developed an immunomagnetic‐based microfluidic device for CTCs capture.^69^ In this work, blood samples were firstly labelled with magnetic nanoparticles functionalized by EpCAM antibodies, and target CTCs were then efficiently captured with high efficiency of 90% when the blood samples flowed through the microfluidic channel closely above arrayed magnets. Huang et al. also reported an immunomagnetic‐based microfluidic system for CTCs isolation from blood samples.^70^ This microfluidic system was operated in a flip‐flop mode in order to reduce the stagnation and non‐specific adhesion of normal blood cells on microfluidic surface in the process of CTCs capture, and high capture efficiency of 90% was finally achieved based on this platform. The same group also developed a versatile immunomagnetic nanocarrier‐based microfluidic platform for capturing CTCs in whole blood.^71^ In this work, CTCs were selectively targeted by EpCAM antibody‐functionalized magnetic nanocarriers and isolated from whole blood samples by magnetic force in a microfluidic chamber with capture efficiency greater than 90%. Chen et al. fabricated a graphite‐coated magnetic nanoparticles microarray chip for CTCs capture and isolation.^72^ The graphite‐coated magnetic nanoparticles with good biocompatibility and stability were synthesized by using the chemical vapor deposition, and the graphite modification on its surface provided functional groups for subsequent antibody labelling to achieve specifically CTCs recognition. Based on this graphite‐coated magnetic nanoparticles microarray chip, efficient CTCs capture from spiked blood samples was successfully achieved even at very low cell concentrations. Similarly, Yu et al. developed a microfluidic system with micropillar array decorated with graphite oxide‐coated and antibody‐functionalized magnetic nanoparticles for CTCs capture and isolation.^73^ Under magnetic field manipulation, the decoration of functionalized magnetic nanoparticles on the micropillars increased the interactions between target CTCs and micropillar surface, and successful CTCs capture from two spiked media was achieved with capture efficiency greater than 70% in culture medium and greater than 40% in blood sample. In another report, Issadore et al. developed a microfluidic chip‐based micro‐Hall detector to capture immunomagnetic nanoparticle‐tagged CTCs from whole blood sample with high efficiency and high‐throughput ability.^74^

High deformability is a distinctive biomechanical property for cells circulating in the peripheral blood, especially for CTCs with larger size than normal blood cells in order to rapidly go through capillaries with small diameters of 6–8 μm and successfully metastasize.^77^, ^78^ Atomic force microscopy (AFM)‐based single cell stiffness study for different cancer cells including lung, breast and pancreatic, have shown that malignancy increase cell deformability at the single cell level although CTC are still more stiffer than blood cells.^40^ Therefore, in addition to size, the unique deformability of CTCs is also a factor that can be used for selective capture and isolation of CTCs. Based on the fact that CTCs are always larger and stiffer than normal blood cells, our group developed a microfluidic device equipped with an array of traps for CTCs capture and isolation from peripheral blood samples.^79^, ^80^ For the structure of trap array, each trap was composed of three pillars with a diameter of 3–4 μm and was arranged in an arc shape with 5 μm distance between pillars. In the process of CTCs capture and isolation, small sized RBCs and WBCs with higher deformability could pass through the 5 μm gaps, while larger CTCs were stuck in the arc‐shaped traps, achieving highly efficient CTCs capture and isolation (Figure 2D). Furthermore, a pre‐filter with 20 μm gap was mounted to prevent larger clumps and debris from clogging up the cell trap area. Highly isolated CTCs can be finally collected from this microfluidic‐based material interface for downstream applications, such as immunological staining and molecular analysis.

In a straight microfluidic channel, fluid shear can generate lateral forces to cause transverse migration of particles.^81^, ^82^ While in a spiral microfluidic channel, an inertial focusing of particle according to its size can be observed due to combination of shear induced life force and Dean drag force, which has been used for size separation of particles, giving some illumination for CTCs isolation by using a spiral microfluidic channel. Separation of CTCs in a spiral channel with rectangular cross‐section has been reported.^83^ Recently, a novel spiral microfluidic device with trapezoidal cross‐section was developed for rapid and efficient label‐free isolation of CTCs from clinically blood samples, by utilizing the inherent Dean vortex flow and inertial lift forces present in the spiral microfluidic channel (Figure 2E).^84^ Compared to conventional spiral microfluidic devices with rectangular cross‐section, the position of Dean vortex core in spiral microchannel with trapezoidal cross‐section can be altered by which larger CTCs will focus and be collected at the inner channel wall outlet while smaller hematologic cells will focus and be removed at the outer wall outlet, thus achieving efficient CTCs isolation and enrichment. Based on this platform, high CTC capture efficiency of greater than 80% were successfully achieved within 8 min from both spiked cancer cells blood samples and clinical peripheral blood samples from patients with advanced stage metastatic breast and lung cancers, providing a powerful tool for CTCs capture and isolation.

## 4 Nanostructured Substrates‐Based Materials Interface for CTC Capture

In tissue engineering and regenerative medicine, nanostructured substrates have been widely employed to mimic the natural extracellular matrix (ECM) and basement membrane.^85^, ^86^, ^87^ These substrates can promote cell attachment due to enhanced local topographic interactions between nanostructures and nanoscale components of the cellular surface such as microvilli and filopodia, thereby assisting the capture and isolation of CTCs.^88^, ^89^ Furthermore, nanostructured substrates can provide more surface area for immobilization of CTC affinity molecules.^90^, ^91^ Hence, nanostructured substrates can be combined with the affinity interactions‐based CTC capture strategy, which can further improve CTC capture efficiency and emerge as a promising platform for isolation, and enrichment of CTCs. In this section, different types of nanostructured substrates‐based platforms for CTCs capture and isolation available will be briefly introduced including nanowires, nanopillars, nanodots, nanofibers, nanosheets, nanotubes and nanopores, and their performances on CTCs capture and isolation summarized in **Table** **2**.

**Table 2:** The performances of nanostructured substrates‐based platforms on CTCs capture and isolation

Nanostructured substrate platform	Capture yield or efficiency	Detection rate or sensitivity	Cell survival rate	Reference
Nanopillar	40%	–	84–91%	92
Nanopillar‐micromixer	95%	–	–	94
Nanowire	67.5%	–	–	93
Nanofiber	45%	–	–	97
Nanosheet	73%	–	–	96
Nanopore	80%			101

**Figure 3:**
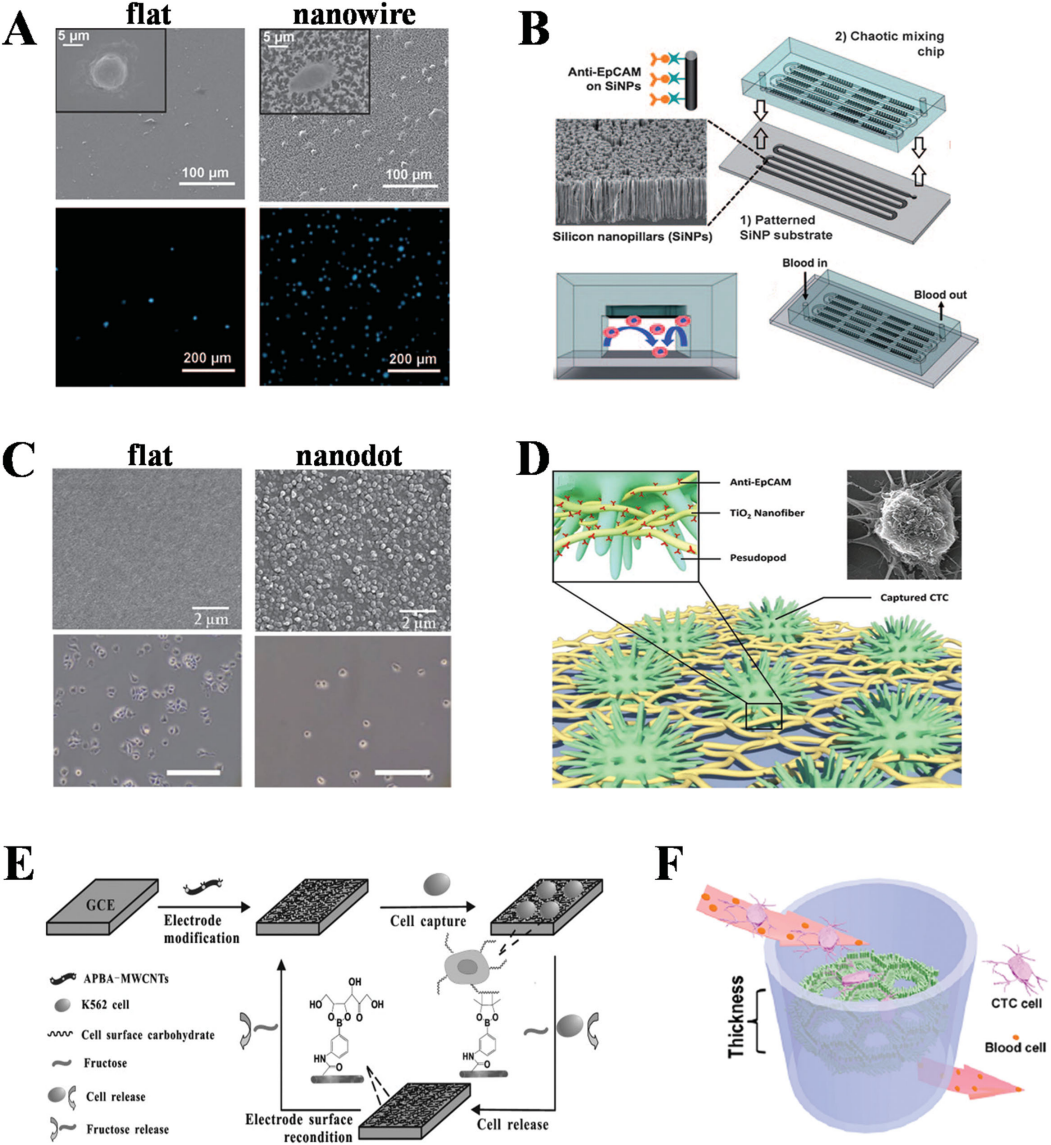
Efficient capture and enrichment of CTCs by using nanostructured substrates‐based material interface. A) Nanopillar substrates. Reproduced with permission.^92^ B) Integrated chaotic micromixer‐nanopillar substrates. Reproduced with permission.^94^ C) Nanodot. Reproduced with permission.^95^ D) Nanofiber. Reproduced with permission.^97^ E) Carbon nanotubes. Reproduced with permission.^99^ F) Nanopore‐based 3D graphene foam. Reproduced with permission.^101^

Inspired by the surface components of cells, the nanowire and nanopillars‐based substrates have been designed and utilized to make use of the surface adhesion of the cells and aid the capture of CTCs in blood samples. For example, Wang et al. firstly utilized anti‐EpCAM‐coated Si nanopillars (SiNPs) substrates to identify and capture CTCs (**Figure** **3**A). Using a wet chemical etching approach, densely packed nanopillars of 100–200 nm in diameter were prepared on silicon wafers; additionally, the length of these nanopillars could be easily controlled by altering the etching times. To test the cell capture efficiency of the SiNPs, cell suspension solution of MCF7 cells (an EpCAM‐positive cell line) was introduced for 1 h into the SiNPs and also flat silicon substrates. It was found that more cells were captured on SiNPs (45–65%) than on flat silicon substrates (4–14%), suggesting that nanopillars are responsible for enhanced cell capture. The performance of SiNPs on CTC capture and isolation was tested in the artifical CTCs blood samples prepared by spiking blood with different densities of tumor cells, and improved capture efficiency of CTCs (40%) was obtained by the SiNPs platforms comparted to some commercially available technologies.^92^ Similar to SiNPs, quartz nanowires (QNWs) was also fabricatied and employed for CTCs capture and quantification in the spiked blood samples to investigate its potential in clinical use.^93^ By increasing the contact frequency between cell and nanopillar substrate, even higher capture efficiency of CTCs could be further achieved. For instance, Wang et al. integrated SiNPs into a microfluidic device with serpentine chaotic micromixers, obtaining a nearly 100% capture efficiency (Figure 3B). To test the performance of this integrated platform for CTC capture, a series of CTC samples was firstly prepared by spiking three kinds of solutions (whole blood, lysed blood, and PBS buffer) with cancer cell lines of MCF7, T24 and PC3, respectively. Under the optimal conditions of flow rate, more than 95% of capture efficiency of target cancer cells was found in all CTCs samples mentioned above by this integrated platform, providing an efficient way for isolation of CTCs and early diagnosis of cancer metastasis.^94^

Nanodot and nanosheet substrates have also been demonstrated to show efficient CTCs capture ability. The dot size and density were controlled by the voltage applied and could be easily reproduced and tuned. Five tumor cell lines of interest were examined and they were either overexpressed with EpCAM antigens or without EpCAM antigens on their cell membranes. Although the aspect ratios of nanodots were small, the efficiency of specific cell capture by anti‐EpCAM conjugated to the nanodots, was enhanced by four to five times in comparison with smooth films (Figure 3C).^95^ The enhancement is most likely due to a synergistic effect from ligand‐receptor interaction, and nanostructure matching of tumor cells and nanodot substrate. In addition to nanodot, nanosheet substrates have also been employed for efficient CTCs capture and isolation. Yoon et al. fabricated a graphene oxide nanosheet substrate‐based device and used it for CTCs capture from blood samples.^96^ After functionalized by CTCs‐selective antibodies, this nanosheet substate‐based device exhibited ability for CTCs capture with efficiency of 73% from blood samples of pancreatic, breast and lung cancer patients at low cell concentrations (3–5 cells mL^−1^).

Inspired by ECM scaffolds, nanofiber‐based substrates have been fabricated and well developed for their efficient CTCs capture efficiency. Different materials such as TiO_2_, and poly(lactic‐co‐glycolic acid) (PLGA) can be electrospun to form desired nanofibers with controllable diameters and lengths. Zhang et al. fabricated TiO_2_ nanofibers of 100–300 nm diameter from a spun composite of titanium n‐butoxide and polyvinyl pyrrolidone (Figure 3D). By coating anti‐EpCAM onto the surface of nanofibers, functionalized platform for CTCs capture was prepared. Using these nanofibers deposited substrates, cancer cells from artificial CTCs blood samples, as well as from whole blood samples of colorectal and gastric cancer patients were reliably captured.^97^ In another report, Hou et al. developed a PLGA‐nanofiber embedded chip (PN‐nanovelcro chip) which not only captured CTCs with high efficiency, but also enabled highly specific isolation of single melanoma cell immobilized on the nanosubstrate.^98^ The PN‐nanovelcro chip was composed of an overlaid PDMS chaotic mixter and a transparent PN‐nanovelcro substrate fabricated by electrospining PLGA nanofibers onto a commercial laser microdissection (LMD) slide and functionalized by a melanoma‐specific antibody. Based on the enhanced local interaction between cell and PLGA nanfibers, target melanoma cells were efficiently captured, and single cell isolation was subsequently isolated by using the highly accurate LMD technique. In order to specifically identify melanoma cells captured on the PLGA nanofibers, a four‐color immunocytochemistry method was also developed in the PN‐nanovelcro chip system.

Nanotubes and nanopores have also been reported to have great potential for CTCs capture and isolation. For example, functionalized multiwalled carbon nanotubes (MWCNTs) films have been successfully used for K562 cells (leukemia cells) capture and electrochemical sensing (Figure 3E).^99^ They prepared the films by covalent coupling between ‐NH_2_ groups in 3‐aminophenylboronic acid (APBA) and ‐COOH groups in acid‐oxidized MWCNTs. Due to the high affinity interacions between the boronic acid groups of APBA and the carbohydrate on cell surface, the K562 cells could be efficiently captured by the APBA‐functionalized MWCNTs films. Compared to bare APBA films, the functionalized MWCNTs one not only exhibited more boronic acid groups for K562 cell recognition, but also provided enhanced local cell‐MWCNTs interactions, which improve K562 cells' adhesion on its surface. Furthermore, the high electrical conductivity of MWCNTs maked the APBA‐MWCNTs film a good electrode for subsequent cell electrochemical sensing, presenting a promising way for efficient capture and highly sensitive electrochemical detection of CTCs. In another report, King et al. explored a method to more efficiently capture leukemic and epithelial cancer cells from flow by altering the nanoscale topography of the inner surface of P‐selectin‐coated microtubes.^100^ In this work, halloysite nanotubes were naturally attached to the inner surface of microtubes to alter their nanoscal topography via a monolayer of poly‐L‐lysine. It was found that the capture efficiency of leukemic cells could be increased by halloysite nanotube coatings and mainly affected by halloysite content and selectin density, making the functionalized microtubes with nanoscale topography a promising platform for enhanced CTCs capture and isolation. In addition to nanotubes, nanopores also open up a new opportunity in CTCs capture and diagnosis. In a recent report, we reported a 3D hierarchical graphene platform that combines microporosity from reduced graphene oxide foam with anti‐EpCAM coated ZnO nanorod array (Figure 3F).^101^ The advantage of this novel composite structure stems from its high density of ZnO nanorods, which increases cell‐substrate contact frequency, as well as its microporosity, which lets through normal RBCs but specifically captures CTCs due to the introduction of EpCAM antibodies. When thickness of the foam reached 5 mm, the cell‐capture yield was more than 80%, indicating its potential CTCs capture capability for clinical blood samples.

## 5 Miniature Nuclear Magnetic Resonance System‐Based Materials Interface for CTCs Capture and Detection

As a novel sensing technology, micro‐nuclear magnetic resonance (*μ*NMR) exploits magnetic resonance technology to detect target labelled with immunospecific magnetic nanoparticles (MNPs), showing great potential in rapid and highly sensitive biodetection.^102^ The typical MNPs used in *μ*NMR are superparamagnetic and have small size (tens of nm), which is different from the conventional magnetic nanoparticles used in immunoseparation. The mechanism of *μ*NMR‐based sensing technique is based on the phenomenon that MNP‐labeled targets exhibit faster relaxation of NMR signals due to local magnetic fields created by MNPs.^103^ By systematically optimization of nanoagents, MNP‐target conjugation method, and NMR detectors, several exquisite *μ*NMR‐based platform have been developed for rapid and sensitive detection of biomolecules including nucleic acids, proteins, bacteria, and tumor cells.^104^, ^105^, ^106^, ^107^, ^108^, ^109^ Compared to conventional biosensing methods, *μ*NMR‐based technique do not need sample purification procedures and can simultaneously achieve target capture and detection, gaining much attention in the fields of CTCs capture and detection. This section will briefly introduce recent developments of *μ*NMR‐based biosening systems and their potential applications in CTCs capture and detection.

**Figure 4:**
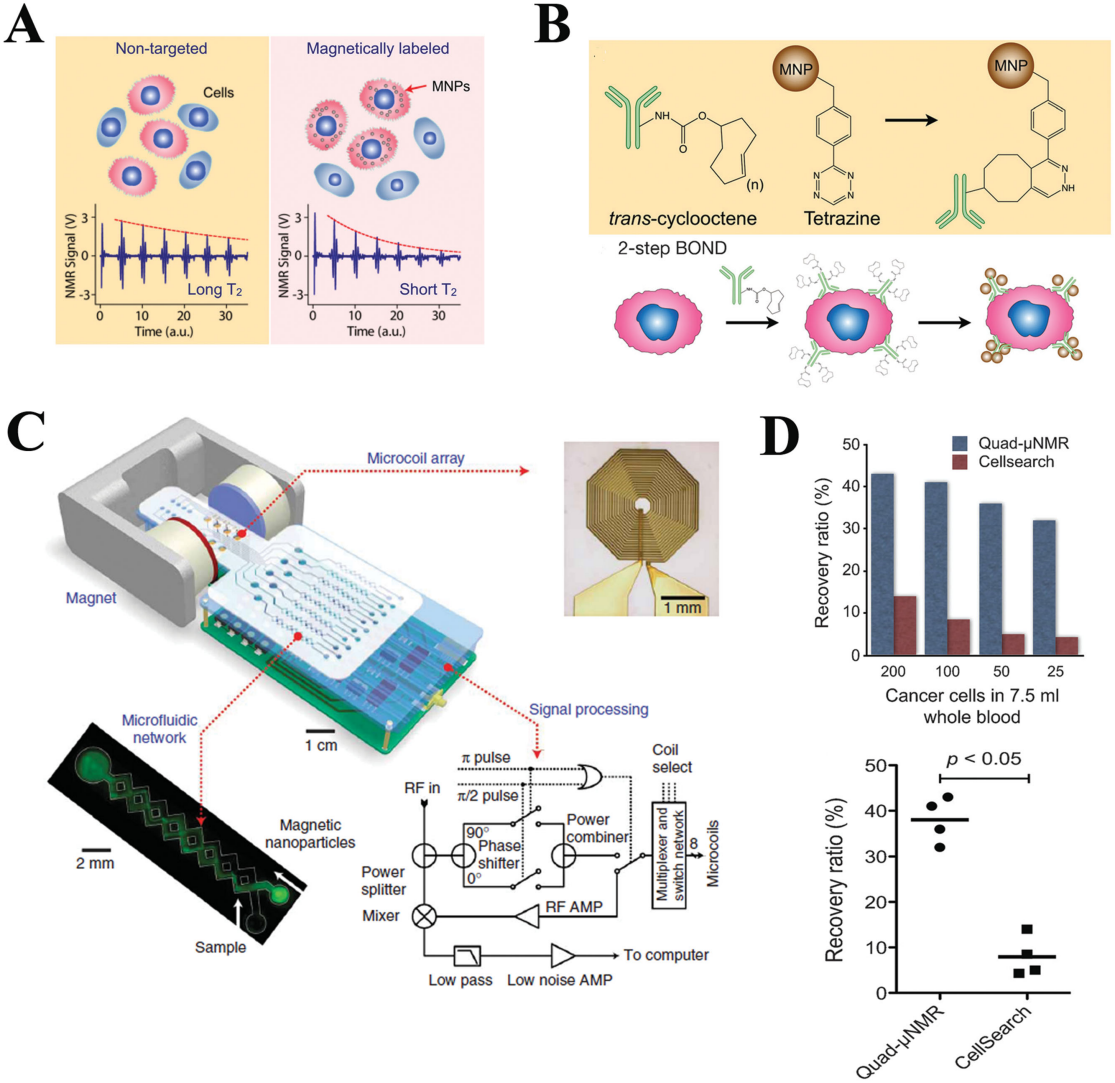
A) Principle of CTC detection based on *μ*NMR system. CTCs tagged with MNPs can accelerate the transverse relaxation of water protons. Compared to the non‐tagged samples (left), the NMR signal will decay faster in time domain (right), providing a sensing mechanism. Reproduced with permission.^102^ Copyright 2008, Macmillan Publishers Ltd. B) Bioorthogonal nanoparticle detection (BOND). The method is based on the Diels–Alder cycloaddition between trans‐cyclooctene (TCO) and tetrazine (Tz). Cells are pre‐labeled with TCO‐antibodies and targeted with Tz‐MNPs. The antibody provides sites for multiple MNP binding. Reproduced with permission.^112^ Copyright 2010, Macmillan Publishers Ltd. C) Typical example of the *μ*NMR system. This system consists of an array of microcoils for NMR detection, microfluidic channels for sample handling, embedded NMR electronics, and a permanent magnet. Reproduced with permission.^102^ Copyright 2008, Macmillan Publishers Ltd. D) CTC deteciton performance comparison between μNMR and CellSearch system. Reproduced with permission.^114^ Copyright 2012, Neoplasia Press.

Based on the “*T2*‐shortening” effect of MNPs in NMR measurements, the detection of CTCs labelled with MNP can be achieved. In NMR measurements, MNPs can produce local magnetic dipole fields with strong spatial dependence, and subsequent destroy the coherence in the spin‐spin relaxation of water protons. Therefore, target labelled with MNP will show shorter transverse relaxation time in NMR measurements, namely the phenomenon of “*T2*‐shortening” effect, compared to target without MNPs label, making detection of CTCs possible (**Figure** **4**A).^102^ For *μ*NMR‐based CTCs detection system, engineering MNPs for high transverse relaxivity and efficient MNP labeling on cells are two important issues which need to be addressed for highly sensitive *μ*NMR sensing.^110^ For the first issue, elemental iron (Fe) exhibiting the highest saturation magnetization and low magnetocrystalline anisotropy among ferromagnetic crystals, may be a good candidate of constituent material for MNPs and it is possible to synthesize superparamagnetic Fe‐MNPs with high transverse relaxivity. Recently Yoon et al. synthesized a new type of hybrid Fe‐MNP with high magnetic moments and transverse relaxivity.^111^ This hybrid particle composed of an elemental Fe core and a protective ferrite shell, showed high transverse relaxivity and stable magnetic properties against oxidation. In addition to the synthesis of MNPs with high transverse relaxivity, strategies for efficient cell MNP‐labeling are also needed for *μ*NMR‐based CTC capture and detection. Recently, a novel labelling strategy for target‐MNPs constructs preparation called BOND (Bioorthogonal nanoparticle detection) was developed by Lee et al.^112^ Based on the reaction between tetrazine (Tz) and trans‐cyclooctene (TCO), namely the Diels‐Alder cycloaddition, BOND can rapidly achieve the covalent binding of MNP to biological targets at room temperature without catalyst (Figure 4B). BOND chemistry has been employed for cell MNPs labelling using a two‐step approach: cell labelling with TCO‐modified antibodies, and the subsequent covalent binding between cell‐antibodies‐TCO and Tz‐loaded MNPs. Since one antibody can be modified by multiple TCO tags without loss of its affinity, multiple attachment of Tz‐MNPs to cells can be subsequently achieved by using the antibodies as scaffolds. Therefore, compared to the method for cell‐MNPs preparation directly using MNP‐antibody conjugates, the two‐step BOND strategy can efficiently amplify MNP‐binding to cells, and then will amplify NMR signals and ultimately enhanced the detection sensitivity, thus showing great potential in *μ*NMR‐based CTC capture and detection.

**Figure 5:**
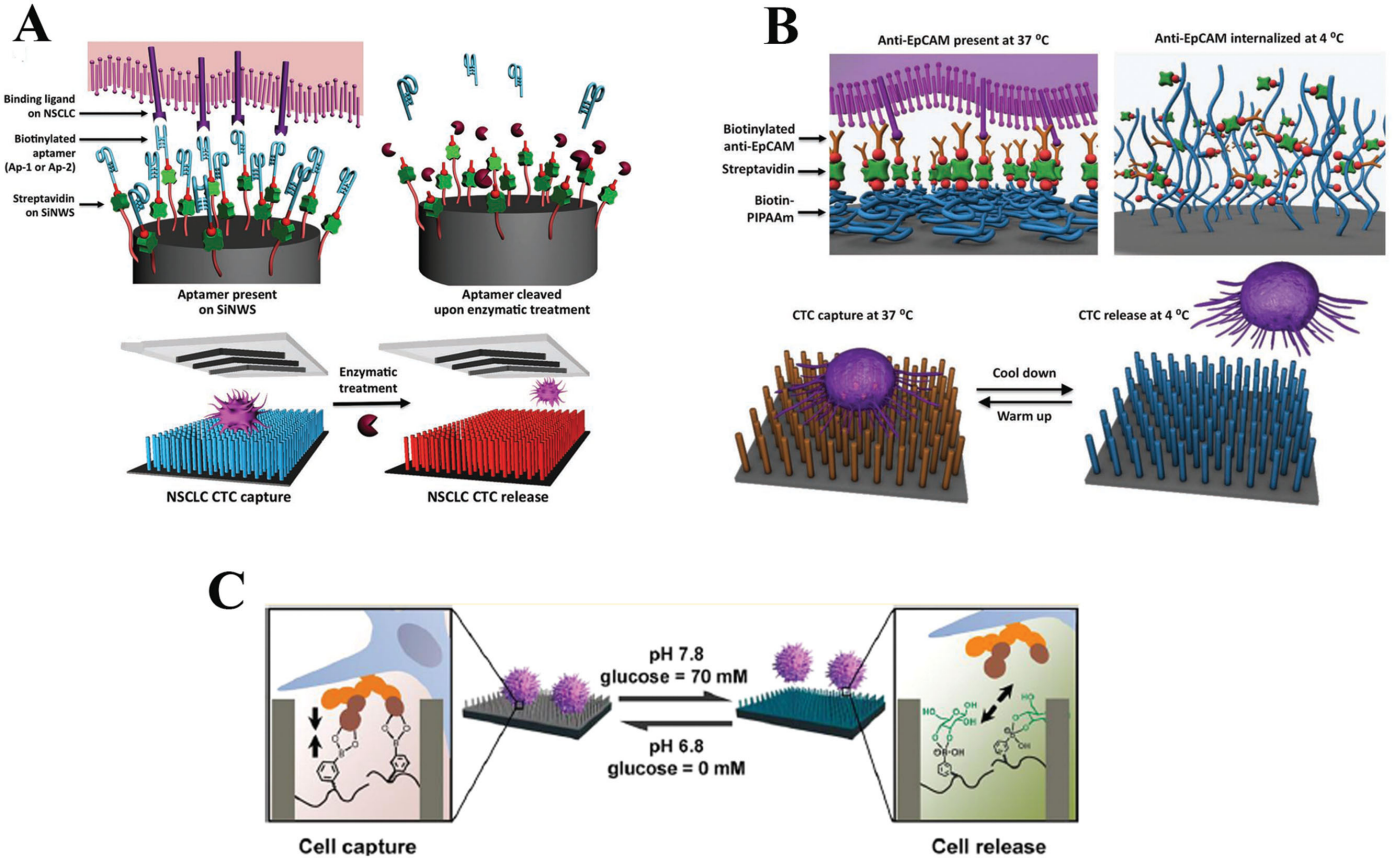
Strategies for controllable CTC release from nanowire substrates. A) Enzymatic treatment. Reproduced with permission.^121^ B) temperature stimulation. Reproduced with permission.^122^ C) pH and glucose stimulation. Reproduced with permission.^123^ Copyright 2013, American Chemical Society.

Till now, different models of μNMR devices with miniaturized system for CTCs capture and point‐of‐care detection have been developed. The miniaturization of μNMR system endows the following advantages: 1) improving detection sensitivity by reducing sample volumes and increasing the concentrations of targets; and 2) generating stronger radio‐frequency NMR magnetic fields due to the use of smaller magnets in miniaturized system. Figure 4C shows a typical miniaturized μNMR device developed for CTCs capture and detection, which included four main parts: microcoils, microfluidic network, custom‐designed NMR electronic and a portable small permanent magnet. On the bottom of this μNMR device, eight planar microcoils with volume of 10 μL were arranged into a 2 × 4 array format to achieve parallel CTCs detection. A microfluidic network was implemented on the top of the microcoils to enable sample handling and distribution. In addition, to compensate for the inhomogeneity of magnetic field generated by the small permanent magnet, a NMR electronic was customarily designed and implemented to allow for spinecho measurement. Appreciating the rarity of CTCs in blood sample and the heterogeneity of CTCs surface biomarkers, quad‐μNMR platform with a quad biomarker “cocktail” (MUC‐1, EGFR, HER2, and EpCAM) for optimal signal and detection was developed for CTCs isolation and detection.^113^ In this cocktail assay, CTCs were simultaneously targeted with TCO‐modified MUC‐1, EGFR, HER2, and EpCAM antibodies and subsequently incubated with Tz‐MNPs to get the quad NMR probes of CTC‐MNPs. For the performance of this quad‐μNMR platform, it was found that an average recovery rate of 38% across the various cell concentrations (200, 100, 50, and 25 spiked cells) tested was obtained, which was higher than the CellSearch system only with an average recovery rate of 9.1%, ultimately leading to higher CTCs detection sensitivity (**Figure** **5**D). Compared to CellSearch system, the quad‐μNMR platform demonstrated 400% fold higher CTCs detection sensitivity, showing great potential in isolation and detection of CTCs with low surface biomarkers expressing cell line, such as the MDA‐MB‐436 with known EMT behavior. Furthermore, CTCs isolation and detection from peripheral blood samples collected from 15 patients with ovarian cancer were successfully and sensitively achieved by using the quad‐μNMR platform, indicating its potential in clinical applications.^114^ In summary, the Quad‐μNMR platform expands the range of CTCs isolation and detection conditions, making it not limited to the case of higher CTCs concentrations such as stage IV, progressive disease, or in patients not pursuing active therapy.

## 6 Approaches for CTC Detection and Identification

Once CTCs are captured and enriched, subsequent detection and identification are needed to investigate their origin and genetic profile from which more valuable insight into the biology of metastasis can be obtained.^115^, ^116^ In μNMR‐based platforms, CTCs detection can be easily achieved by analyzing the NMR signals of MNP‐labelled target tumor cells without prerequisite isolation and enrichment processes. Hence, this section focuses on the approaches of CTCs detection and identification used in microfluidic‐ and nanostructured substrates‐based platforms, which mainly involve immunological and molecular methods.

Most CTCs immunological identification assays use different fluorescent dyes to simultaneously stain cytokeratins (positive marker for epithelial tumor cells) and leukocyte antigen CD45 (exclusion marker). The cell staining process is always carried out in situ in microfluidic‐ and nanostructured substrates‐based platforms. For example, in size filtration‐based microfluidic system with microcavity arrays, two fluorescent immunological probes of FITC‐labelled anti‐CD45 antibody and PE‐labelled anti‐EpCAM antibody were employed to detect and identify CTCs captured on the microcavity arrays.^59^ Similarly, in a recent study, CTCs captured on the aptamer‐functionalized SiNWs substrates were distinguished from non‐specifically trapped WBCs by using a three‐color immunological method based on FITC‐labeled anti‐EpCAM, Cy5‐labeled anti‐CD45, and DAPI nuclear staining.^117^ Most CTCs molecular identification methods use DNA testing techniques such as polymerase chain reaction (PCR) and restriction fragment length polymorphism (RFLP) to analyze the specific DNA or mRNA of CTCs enriched.^117^, ^118^ PCR‐based analysis technique are the most widely used molecular method for CTCs detection and identification. For example, Devriese et al. used PCR‐based technique to analyze a panel of gene marker of CTCs including cytokeratin 7, cytokeratin 19, human epithelial glycoprotein and fibronectin 1 for selective identification and detection of CTCs in non‐small lung cancer, and achieved sensitivity of 46% and a specificity of 93% in 46 cancer patient.^119^ In another study, Hoe et al. used PCR to do the single CTC genotyping for a key melanoma drug target mutation after capturing CTCs using a nanofiber‐embedded microchip.^98^

For approaches of CTCs detection and identification, there are still two factors needed to be taken into consideration. Firstly, heterogeneity among CTCs is a problem that can not be ignored for CTCs detection and identification, which makes cell‐to‐cell variations occur in same cancers and only a very small fraction of CTCs that may eventually acquire the ability to seed the metastatic tumor. Hence, how to characterize molecular, phenotypic and functional difference of CTCs at the single‐cell level is a critical problem that need to be reviewed. Lee et al. reported a laser scanning cytometry‐based method for CTCs detection and identification, by which automated and rapid characterization of physical and functional cellular properties such as size, shape and signaling proteins of CTC at the single‐cell level was quantitatively achieved.^93^ In another report, by combining the PLGA nanofiber substrate with the laser microdissection (LMD) technique, an exquisite platform for CTCs capture and detection was successfully developed by Tseng group.^98^ In this work, based on an LMD microscope, captured CTCs could be cut out and harvested at the single‐cell level, making subsequent single‐cell molecular analysis possible. In addtion to the problem of CTCs heterogeneity, controllable release strategy of CTCs after capture are also needed for subsequent detection and identification of CTCs. For microfluidic CTCs capture platforms, magnetic‐based release strategy is the main method widely used in CTCs molecular identification process.^70^, ^71^, ^72^, ^73^, ^120^ For example, in a recent study, Yu et al. developed a microfluidic platform with micropillar array decorated with magnetic nanoparticles for CTCs capture with efficiency of greater than 70% when the magnetic field was applied, and the captured CTCs could be released with high efficiency of 92.9% upon the removal of applied magnetic field.^73^ Moreover, it was found that 78% of the released CTCs was viable, laying a solid foundation for subsequent molecular analysis. For nanostructured substrates‐based CTCs capture platforms, different CTCs release strategies have been reported, which can be divided into three categories: enzymatic treatment, temperature, and pH and glucose dual stimulation. Detailed explanation for the three strategies will be given below. Enzymatic strategy for controllable CTC release after capture was firstly demonstrated for the SiNWs‐based CTCs capture platform by Shen et al. (Figure 5A). In their work, by modified the SiNWs substrates with CTC selective DNA aptamers generated via Cell‐SELEX process, a new integrated SiNWs‐microfluidic chaotic mixture‐based CTCs capture platform was fabricated. This aptamers‐functionalized platform could not only achieve efficient CTCs isolation from blood with improved capture efficiency compared to the conventional EpCAM‐functionalized platform, but also realize controllable CTCs release after capture by nuclease treatment.^121^ In another report, Hou et al. developed an exquisite platform with CTC capture and on‐demand release ability based on thermally responsive Poly(*N*‐isopropylacrylamide) (PNIPAAm) brushes‐modified SiNWs substrate (Figure 5B). This platform exhibited superior performances in capturing cancer cells with high efficiency at 37 °C, and releasing the captured cancer cells with great viability and retained functionality at 4 °C.^122^ Recently, Jiang et al. developed a pH and glucose‐responsive strategy for CTCs release after capture based on poly(acrylamidophenylboronic acid) (polyAAPBA) brush‐grafted aligned SiNWs substrate (Figure 5C). By precisely controlling pH and the glucose concentration in CTCs samples, reversible capture and release of CTCs could be successfully achieved with dual‐responsive performance. Specifically, the polyAAPBA‐grafted SiNWs substrate changed its state from cell‐adhesive to cell‐repulsive with the increase of pH from 6.8 to 7.8 in the presence of 70 mM glucose. Under the condition of pH 7.8, the polyAAPBA‐grafted SiNWs substrate became glucose responsive, which could capture targeted cells in the absence of glucose and release them in presence of 70 mM glucose. The dual‐responsive capture and release of CTCs on this polyAAPBA‐grafted SiNWs substrate is noninvasive with higher cell viability of 95%.^123^

## 7 Challenges and Future Perspectives

In the previous sections, we described various advanced materials interface mainly based on microfluidics, nanostructured substrates, and micro‐nuclear magnetic resonance systems for CTCs capture and detection. Although promising results have been achieved by these interfaces in terms of capture efficiency and detection sensitivity, most of them still remain in the laboratory level and little of them unequivocally shows clinical validity and utility.^124^ Heterogeneity among CTCs is a problem that cannot be ignored for CTCs isolation. CTCs always express variable biomarkers on their membrane, which affects their morphology and characteristics and makes cell‐to‐cell variations occur in same cancers, same patient or even within a single blood draw.^125^, ^126^, ^127^ Therefore, efficient CTCs capture and isolation are challenging due to this heterogeneity of CTCs, indicating that not a single cell surface biomarker can confidently be used for total CTCs isolation. That is why current widely used EpCAM antibodies‐based CTCs capture systems do have concerned limitations in clinical applications. Similarly, the immunological capture methods predominantly used in nanostructured substrates and nuclear magnetic resonance‐based platforms do have similar concerns. By exploiting the inherent unique biophysical properties of CTCs, label‐free microfluidic strategies seems to have greater potential in clinical capture and isolation of CTCs compared to the currently utilized biomarker‐based immunological methods by which only a subset of CTCs expressing the selected surface markers are isolated. However, the label‐free microfluidic approach might also introduce false positives results in clinical CTCs capture and isolation by capturing cells that may not directly originate from the primary tumors.^128^ In addition, captured CTCs by some label‐free microfluidic platforms are no longer intact after being subjected to shear forces, thus making subsequent CTCs identification and detection difficult.

Beyond the issue of capturing and isolating CTCs, achieving a better understanding of the molecular characteristics of CTCs is also important from which new biomarkers for efficient CTCs capture and isolation can be discovered. However, traditional CTCs molecular analysis is always performed by using large ensembles on the order of 10^3^–10^6^ cells, thereby only giving the average genotypic or phenotypic characteristics of the cell population. In addition, the way the isolated cells are cultured to expand CTC numbers is not recommended, given that cancer cells have a feature that modifies their characteristics to survive when the surrounding microenvironment is changed.^129^ Single cell analysis has been widely used to explore cellular heterogeneity in gene and protein expressions responsive to environmental change and chemotherapeutic stimuli, providing an efficient way for CTCs heterogeneity study.^130^, ^131^ Therefore, rationally designed platforms with highly efficient CTCs capture and single cell evaluation ability are expected to be promising tools for future CTCs study. Learning from the experiences of previous literatures reviewed above, single‐cell evaluation technique such as laser scanning cytometry and microfluidic systems with nanostructure arrays may be a good candidate for the expected objective, given that microfluidic techniques offer efficient label‐free CTCs separation while the existence of nanostructure arrays confine the CTCs migration and enhance the interactions between target CTCs and microfluidic surface. Furthermore, in order to achieve highly efficient CTCs capture, multi‐biomarkers of CTCs (e.g., EpCAM, HER2, EGFR, and MUC‐1) can be patterned to the different regions of microfluidic system, by which CTCs with different biophysical and biochemical properties can be isolated. Moreover, with the help of single‐cell evaluation technique, captured CTCs can be further sensitively detected and characterized by immunological and molecular methods at the single‐cell level.
